# Beyond maximum density: multi-parameter insights into Scots pine climate sensitivity

**DOI:** 10.1007/s00468-025-02681-3

**Published:** 2025-09-22

**Authors:** Inga K. Homfeld, Frederick Reinig, Edurne Martinez del Castillo, Max C. A. Torbenson, Oliver Konter, Rob Wilson, Paul J. Krusic, Neil J. Loader, Hakan Grudd, Emily Reid, Kayleigh Letherbarrow, Jan Esper

**Affiliations:** 1 Department of Geography, Johannes Gutenberg University, 55099 Mainz, Germany; 2 Global Change Research Institute, Czech Academy of Sciences, 603 00 Brno, Czech Republic; 3 School of Earth & Environmental Sciences, University of St. Andrews, St Andrews, UK; 4 Department of Geography, University of Cambridge, Cambridge, UK; 5 Department of History, Stockholm University, Stockholm, Sweden; 6 Prifysgol Abertawe / Swansea University, Swansea, UK; 7 Swedish Polar Research Secretariat, Abisko Scientific Research Station, Abisko, Sweden

**Keywords:** Wood density, X-ray densitometry, *Pinus sylvestris*, Climate change, Scotland, Sweden

## Abstract

**Key message:**

Climate sensitivity of *Pinus sylvestris* has changed in minimum density while maximum density remains mostly stable, suggesting the use of additional density parameters could help detect response changes.

**Abstract:**

As one of Eurasia's most widely distributed conifer species, *Pinus sylvestris* L. is frequently used in dendroclimatological reconstructions based on tree-ring width (TRW) and maximum latewood density (MXD). However, the climatic signals of additional parameters such as earlywood/latewood density (EWD/LWD) or minimum density (MND) are often overlooked, leaving their skill unexplored. Here, we investigate the growth responses of multiple *P. sylvestris* tree-ring parameters to ongoing climate change at two sites with contrasting climatic conditions using well-replicated density data from Scotland and Sweden. Correlations with mean, minimum, and maximum temperatures are strongest for LWD and MXD at both sites, with coefficients ranging from 0.5 to 0.7 for July, August, and the June–August season (*p <* 0.05). A significant (*p <* 0.05) negative correlation between MND and July temperatures was identified in the Swedish Torneträsk (TOR) data (*p <* 0.05), which diminished since the late twentieth century. A comparable inverse MND temperature signal and change into the twenty-first century is not reflected in northern Scotland’s overall wetter and warmer site, suggesting a fundamental physiological change in tree-ring formation under global warming. A shift in the sensitivity of tree growth at northern European sites could reduce the effectiveness of proxies from such locations, posing implications for high-resolution climate reconstructions.

**Supplementary Information:**

The online version contains supplementary material available at 10.1007/s00468-025-02681-3.

## Introduction

Scots pine (*Pinus sylvestris* L.) has a broad distribution that extends from Western Europe to Eastern Siberia and from Southern Europe to Fennoscandia, reaching into the Arctic Circle (Houston Durrant et al. 2016). Consequently, the species can thrive under a wide variety of climatic conditions, with limiting factors that range from drought constraints in the southern regions to temperature restrictions at higher elevations and latitudes. These varied climate-growth relationships, combined with the species’ extensive range, have made Scots pine a common species for dendroclimatic analyses. Consequently, the species has been successfully used as a proxy for temperature (Schweingruber et al. 1979, 2008; Hughes et al. 1984; Briffa et al. 1992; Büntgen et al. 2011; Esper et al. 2012a), drought (Büntgen et al. 2013; Camarero and Hevia 2020; Seftigen et al. 2020; Camarero et al. 2021), precipitation (Ruiz-Labourdette et al. 2014; Belokopytova et al. 2018), vapour pressure deficit (Treydte et al. 2024), cloud cover/sunshine (Young et al. 2012, Loader et al. 2013), past volcanic activity (D’Arrigo et al. 2013) and orbital forcing (Esper et al. 2012b). TRW is the most commonly used parameter in dendroclimatic studies due to its low cost and minimal time investment for data generation (Briffa et al. 2002; Esper et al. 2012b). By contrast, producing density measurements is more time-consuming and requires highly specialized equipment (Schweingruber 1988). Nevertheless, MXD consistently captures a stronger climatic signal than TRW, particularly regarding temperature from conifers growing at high-latitude or high-elevation (i.e., temperature-limited) sites (Briffa et al. 1998; Grudd 2008; Esper et al. 2016). Due to MXD’s superior performance, the other density parameters that are automatically generated at the same time are commonly excluded from any analyses (Björklund et al. 2017). However, evidence suggests these additional density parameters have significant potential as climate proxies. For instance, in drought-prone environments, studies have shown that minimum density (MND) can serve as a proxy for drought severity (Camarero et al. 2014; Camarero and Hevia 2020). Similarly, a considerable influence of spring precipitation on MND was observed in three conifer species, including Scots pine from cold-dry sites in Spain and Russia (Camarero et al. 2017). Meanwhile, strong associations of MND with maximum temperature and drought have been shown for parts of China (Liu et al. 1997; Song et al. 2025), and earlywood density (EWD) has been successfully used for a maximum temperature reconstruction (Chen and Yuan 2014). Compared to the numerous, often millennial-length MXD-based temperature reconstructions (Gouirand et al. 2008; Esper et al. 2012a; McCarroll et al. 2013; Matskovsky and Helama 2014; Schneider et al. 2015), the additional density parameters of MND, total tree-ring density (TRD), earlywood density (EWD), and latewood density (LWD) remain largely unexplored. With the emergence of wood anatomical studies, renewed efforts are underway to enhance our understanding of wood formation processes and to reassess the additional value of these other tree-ring density parameters (Björklund et al. 2019). Such advances provide the essential groundwork needed to better understand tree-ring density variability and the role of associated climatic influences (Björklund et al. 2017).

Northern Scandinavia has for many years been a focal point for dendroclimatic analysis of Eurasian Scots pine due to factors relating to accessibility and the species’ high sensitivity to temperature in far northern latitudes. This has led to the creation of several millennium-long MXD chronologies (Schweingruber 1988; Briffa et al. 1992; Grudd et al. 2002; Grudd 2008; Helama et al. 2010; Esper et al. 2012a), as well as many TRW chronologies—the longest of which spans the past seven millennia (Briffa et al. 1990; Grudd et al. 2002). These TRW records provide the basis for additional dendrochronological analyses, such as those based on latewood blue intensity (LWBI; Björklund et al. 2014; Seftigen et al. 2020), isotopic accumulation (Seftigen et al. 2011; Loader et al. 2013; Torbenson et al. 2022), and wood anatomical studies (Björklund et al. 2023). Most studies focus on summer temperature, with reconstructions targeting a range of seasonal windows, from July–August to April–August, depending on the parameters analyzed.

Fewer reconstructions exist in Scotland, but early research demonstrated a strong influence of summer temperatures, with the first reconstruction using tree-ring records from the Highlands, targeting July–August temperatures from Edinburgh (Hughes et al. 1984). More recently, combined TRW and blue intensity (BI) samples were utilized to produce the region's longest reconstruction of July–August temperatures, covering more than 800 years (Rydval et al. 2017).

TRW/BI-based spatial gridded reconstructions found evidence of systematic “divergence” (D’Arrigo et al. 2008; Esper and Frank 2009) in West Scotland but not in East Scotland, where the rising temperatures during the late 20th and early twenty-first centuries were accurately captured (Rydval et al. 2017). Meanwhile, early reconstruction efforts reported incidents of divergence in TRW and MXD records from the Torneträsk region (Briffa et al. 1998), later attributed to methodological causes (Melvin et al. 2007; Grudd 2008). Within the last decade, multiple studies from Northern Scandinavia incorporating material from Torneträsk have focused on minimizing disagreement between published records by improving chronology construction methods and exploring new proxies or combinations of existing proxies (Linderholm et al., 2015; Björklund et al. 2020). However, disagreement on centennial and longer timescales remains, while the recent temperature rise is faithfully reproduced without divergence (Björklund et al. 2020; Linderholm et al., 2015; Esper et al. 2012b).

Open questions remain regarding the role of signal stability across sample ages (Esper et al. 2008). While results differ between species (Carrer and Urbinati 2004; Esper et al. 2008) and between measurement parameters, MXD appears to be less affected than TRW (Konter et al. 2016) by including multiple age classes. Differing climatic responses of young trees from those of old trees, referred to as climate signal age effects (CSAE, Esper et al. 2008), have been studied for different species and regions (Wilson and Elling 2004; Esper et al. 2008; Fish et al. 2010; Dorado Liñán et al. 2012). Results are inconclusive as both decreasing and increasing climatic responses have been found for young tree rings (Esper et al. 2016; Konter et al. 2016; Ljungqvist et al. 2020).

Here, we investigate the skill of five tree-ring density parameters (TRD, EWD, MND, LWD, MXD) at two *Pinus sylvestris* sites near the northern distribution limit for temperature reconstructions. We evaluate whether tree age influences climate signal stability across the various density parameters by utilizing the uneven age structure at both sites. This approach offers a detailed characterization of climate control on density beyond MXD and improves our understanding of how changing climatic conditions may impact *Pinus sylvestris* growth at the species’ northern distribution limit.

## Materials and methods

**Fig. 1:**
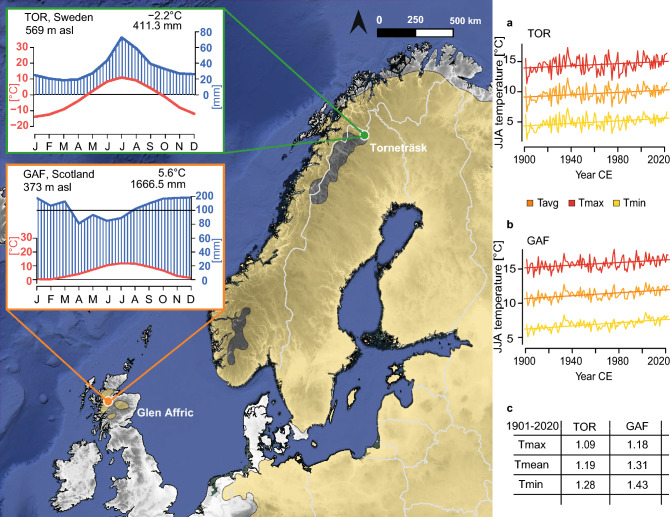
Distribution range of *Pinus sylvestris* in northern Europe (yellow shading) and location of Glen Affric in Scotland (orange dot) and Torneträsk in Sweden (green dot). Insets display the site's climate diagrams based on the nearest grid point CRU data from 1961 to 1990. **a-b**, Minimum, mean, and maximum JJA temperature trends at TOR and GAF from 1901 to 2020, and **c**, associated total changes over 1901–2020 for each site and record

The first study site, Torneträsk (TOR), is close to lake Torneträsk (68.21N, 19.75E, 392 m). A second site was established in Glen Affric (GAF), Scotland (57.27N, -4.89E, 315 m). These two sites represent the northern- and westernmost distribution limits of native Scots pine in northern Europe (Fig. 1). Each site can be characterized as a mixed-species stand with Scots pine as the dominating species and well-developed soil horizons at both sites. Stands are relatively open at both sites with northeastern (TOR) and northern (GAF) orientation and small elevational differences between sampled trees of 3 m at TOR and 5 m at GAF. These factors suggest that the two sites have similar ecological conditions with negligible impact on density.

### Instrumental data

Gridded monthly mean, minimum, and maximum temperatures, along with total precipitation data, were retrieved for the nearest grid point to each site (TOR 68.21 N 19.75 E, GAF 57.27 N -4.89E) from the 0.5° gridded CRU TS 4.08 climate dataset (Harris et al. 2020). The self-calibrated Palmer Drought Severity Index (scPDSI; Wells et al. 2004) was obtained for the closest grid point from the CRU scPDSI 4.08 dataset. All datasets were accessed through the Royal Netherlands Meteorological Institute’s (KNMI) Climate Explorer (Trouet and Oldenborgh 2013), covering the last 120 years from 1901 to 2020, and were analyzed for potential trends using linear regression. Climatic conditions at the two sampling sites display contrasting hydroclimatic variability, with consistently wetter conditions at GAF, where the driest month (April) receives more precipitation (81.1 mm) than the wettest month at TOR (July, 72.7 mm). Peak precipitation occurs during summer at TOR, while GAF experiences the highest precipitation during winter, reaching 193.9 mm in December. Mean monthly temperatures are similar during the summer at both sites, with the warmest month being July at TOR (11 °C) and GAF (11.8 °C). The largest differences are observed during the winter season, where GAF experiences milder mean temperatures that never drop below freezing (lowest at 0.2 °C in February), while the monthly mean temperature at TOR is consistently below zero between October and April (lowest -13.9 °C; Fig. 1).

### Tree-ring data and detrending

At each site, 50 mixed-age trees were selected, and two 5-mm core samples were extracted using Haglöf increment borers. The samples were surfaced using a sliding microtome, and ring widths measured to a precision of 0.001 mm using LINTAB measuring devices (RinnTech, Heidelberg, Germany) and TSAP software (RinnTech 2003). The statistical crossdating of the measurements was performed using the program Cofecha (Holmes 1983). Following ring-width measurement and cross-dating, all samples were prepared for wood density analysis according to standard X-ray densitometry procedures (Schweingruber et al. 1978). Density data were generated using a Walesch X-ray densitometer (Dendro2003, Walesch Electronic, Switzerland), which measures absolute wood densities in g/cm^3^ at resolutions appropriate for the narrow rings expected from tree-line sites (Schweingruber and Briffa 1996; Seftigen et al. 2020). The Walesch software facilitates the extraction of not only MXD but also several other parameters: TRD, EWD, MND, and LWD. Using the Arstan software (version 49; Cook et al. 2017), an age-dependent spline (ADS) with an initial 50-year spline stiffness was selected for detrending all parameters (Melvin and Briffa 2008). All data underwent power transformation (Cook and Peters 1997), and changes in variance were adjusted using the rbar (average interseries correlation; Cook and Kairiukstis 1990)-weighted spline method to account for the effects of replication changes and covariance over time (Frank et al. 2007). Standardized indices were computed as residuals, then averaged using a biweight robust mean (Cook 1985), resulting in five parameter chronologies per site correlated with each other to evaluate relationships between the different parameters. To assess potential negative impacts of uneven sample age, each dataset was divided based on sample age, creating groups of young (< 100/70 years for TOR/GAF) and old (> 100/70 years for TOR/GAF) samples, referred to as *young* (52/38 series for TOR/GAF) and *old* datasets (48/61 series for TOR/GAF). The *young* and *old* datasets were detrended in the same manner as the previously detailed chronologies, which hereafter are referred to as the *full* chronologies. Additionally, the *young* datasets were also detrended using a 30-year fixed spline with the same variance stabilization and chronology calculation as detailed above, for a comparison with 30-year spline detrended instrumental data. A minimum replication of five samples was utilized for all analyses of the resulting site-averaged chronologies. Multiple measures describing chronology quality, as well as all further analyses, were conducted in R (R Core Team 2022) using the packages treeclim (Zang and Biondi 2015) and dplR (Bunn 2008).

### Assessment of chronology climate signals

Bootstrapped climate-growth correlations for the *full* chronology versions of all density parameters were calculated for individual months from the previous June to the current September and across multiple summer seasons, utilizing the five climate variables over the maximum overlap period (1902–2020). Additionally, two 40-year periods were used to assess the impact of sample age: 1980–2020 for both the *young* and *old* chronologies to ensure comparability. The earlier period from 1940 to 1980 was chosen for the *old* chronologies to evaluate signal strength before incorporating the *young* samples. The temporal stability of the climate signal was assessed using bootstrapped 31-year moving Pearson correlations, which were incrementally adjusted one year at a time for the *full* chronologies. The initial significance levels for the climate correlations were adjusted using a Bonferroni correction to avoid overestimation related to multiple comparisons (Bonferroni 1937; Torbenson et al. 2025). This adjustment considers the number of single months, seasons, and parameters.

## Results and discussion

### Characteristics of density parameters

Five well–replicated density chronologies were established for both GAF and TOR (series replication exceeds five from 1796 to 1741 onwards, respectively), revealing minor differences in chronology coherence but comparable relationships among the parameters. Statistical comparisons between the five parameters at both sites show high agreement between MXD and LWD, while MND, EWD, and TRD are less correlated with MXD and with each other (Table S1). TRD, LWD, and MXD demonstrate lower 1st-order autocorrelation than EWD and MND (Table S1), confirming a greater influence of growing conditions during the previous year on EWD and MND (Björklund et al. 2017). Significant correlations (*p* < 0.05) exist between parameters, particularly for EWD and LWD and their respective extreme values, MND and MXD (0.90–0.99), as well as between EWD/LWD/MND/MXD and TRD at each site (0.52–0.86; see Table S1 for details). A notable difference is the weak negative association of MND with LWD and MXD at TOR, which is statistically insignificant, in contrast to a significant positive association with both at GAF (Table S1). Overall, the TOR chronologies exhibit greater mean series intercorrelation (0.41–0.64 vs. 0.34–0.48) and lower autocorrelation (AR1; 0.18–0.37 vs. 0.31–0.43) compared to the GAF chronologies (Table S1). The slightly lower statistics of the GAF chronologies could reflect disturbances related to the human impact in the region (Rydval et al. 2016). Nonetheless, the differences between the chronologies from the two sites are small enough to suggest a similar sensitivity to local climatic (temperature) conditions during tree-ring formation (see Fig. S1 for regional curves).

### Climate response and temporal stability

**Fig. 2:**
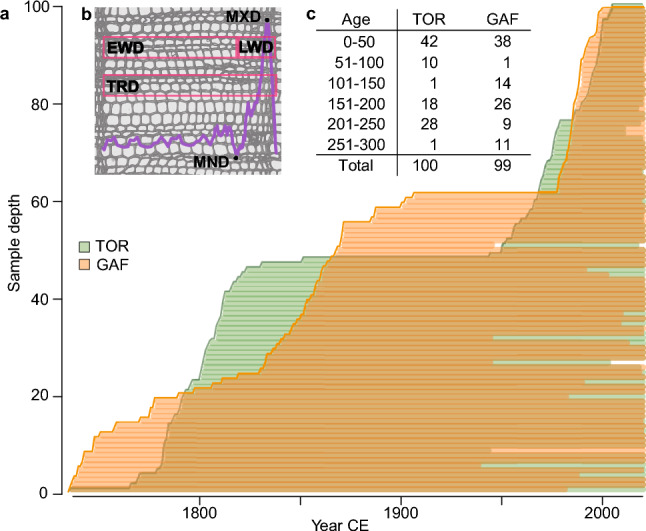
Characteristics of the two *Pinus sylvestris* sites. **a**, Sample replication of TOR (green) and GAF (orange). Each bar represents a single series. **b**, Illustration of the density parameters in a single tree ring, and **c**, comparison of the age distribution of both sites in 50-year bins

These strong similarities are further supported by climate correlations at both sites, which reveal significant (*p* < 0.05) positive relationships of LWD and MXD with monthly mean temperatures from June to August and all seasonal averages. However, the correlations weaken in September (Fig. 2). These results align with previous studies from both sites, which have reported strong and significant MXD correlations with summer temperatures (Büntgen et al. 2011; Esper et al. 2012a; Rydval et al. 2017). The differences between LWD and MXD are minor in TOR and GAF, with the highest correlations between MXD and JJA mean temperature (0.76 vs. 0.77 and 0.69 vs. 0.70, respectively). Including May or even April in the seasonal mean, which some previous studies have focused on (Briffa et al. 1992), yielded no improvement. These patterns hold for correlations with maximum and minimum temperatures (Fig. S2). One difference is the significant correlation of GAF TRD, EWD, and MND with February to April mean temperature, which is not found for TOR, indicating an earlier start of the growing season at GAF related to the stronger oceanic influence at this site (Swain 1987). This difference can also be found between BI parameters from the two regions (Seftigen et al. 2020; Rydval et al. 2014). A persistent, significant negative correlation between local mean July temperature and TOR MND and EWD (-0.53 and -0.30, respectively; Fig. 2a) is not found in the GAF MND and EWD but has been noted for *Pinus sylvestris* samples from Northeastern Finland (Björklund et al. 2020). Instead, the GAF EWD and MND show non-significant, weak positive correlations with mean July temperatures (Fig. 2b). Correlations with precipitation and scPDSI data provide no further clarification of the conflicting MND results, as parameters at both sites show similar responses (Fig. S3). The slightly stronger responses of the TOR MND to scPDSI remain low, with coefficients below 0.3 (Fig. S3 c, d). Across the analysed period, the TOR MND signal for July and JJA temperature indicates an abrupt weakening beginning in the mid-1980s after being relatively stable over the first 60 years (Fig. 2c). At both sites, the MXD signal remained stable over much of the past century for mean July and mean JJA temperature with slight decreases after 1980, particularly for July, and less so for JJA (Fig. 2c, d). While these declines could be signs of divergence, they might also result from an uneven sample age structure.

### Influence of age composition

**Fig. 3:**
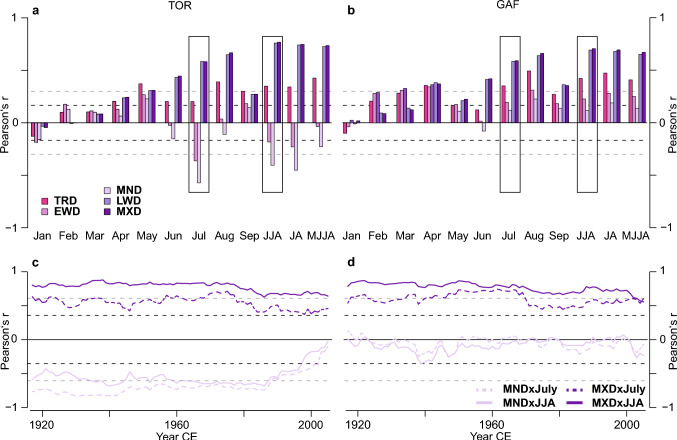
Temperature sensitivity of tree-ring density parameters. **a-b**, Correlations of the TOR and GAF chronologies with monthly and seasonal temperatures from 1902 to 2020. **c-d**, 31-year moving correlation of the TOR and GAF MND (light pink) and MXD (purple) chronologies against July (dashed) and June-July–August mean temperatures (solid) from 1902 to 2020. Dashed black lines indicate *p* < 0.05 significance, and dashed grey lines indicate significance adjusted using Bonferroni correction

**Fig. 4:**
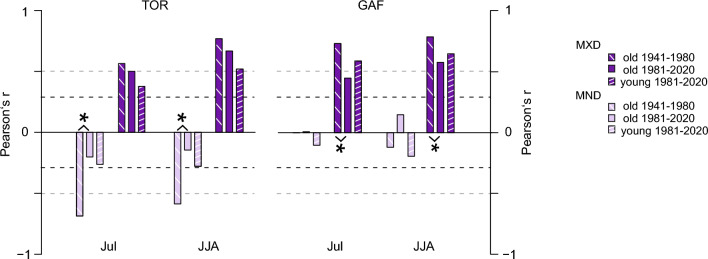
Influence of tree age on climate signals. Correlation of MND (light pink) and MXD (purple) ADS-chronologies, created from older and younger samples, with July and June-July–August (JJA) temperatures. Dashed black lines indicate *p* < 0.05 significance and dashed grey lines indicate significance adjusted using the Bonferroni correction. Asterisk indicating significant differences between periods

Analysis of the age structure for both datasets reveals an uneven distribution with clusters of 52 (38) samples shorter than 100 (70) years for the TOR (GAF) dataset of which 42 (38) are below even 50 years (Fig. 3). Historically, dendroclimatological studies have focused on even-aged datasets to maximize chronology length and signal stability utilizing mature and old trees (Nehrbass-Ahles et al. 2014). However, some studies indicate that even-aged datasets can introduce bias to reconstructions (Nehrbass-Ahles et al. 2014; Szeicz and MacDonald 1994). Our analyses of the *young* and *old* datasets reveal different climate responses between MXD and MND, which are in line with results for the *full* chronologies (see Fig. S4, S5 for chronologies and temperature comparison). Analysis of chronology statistics shows lower coherence in the GAF *young* chronologies compared to the full, while TOR *young* chronologies are similar results to the corresponding *full* chronologies (Table S2). Signal strength for July correlations between the TOR *young* and *old* MXD chronologies shows small, statistically insignificant differences for the 1981–2020 period at 0.38 and 0.49 with JJA correlations at 0.53 and 0.67, respectively. The differences between the correlation of the GAF *young* and *old* MXD chronologies with July temperatures are on a similar scale, also statistically insignificant but flipped, with the *young* chronology correlating stronger at 0.58 than the *old* chronology at 0.45, similar for JJA at 0.65 and 0.57, respectively. Results are mixed for the MND chronologies and vary greatly between the sites and periods. The differences between correlations of the TOR *young* and *old* MND chronology with July and (JJA) mean temperatures are minor at −0.27 (−0.28) and −0.19 (−0.14) for the late period (1981–2020). While there is almost no correlation between either the *young* or *old* GAF MND chronologies and July mean temperature, values diverge for JJA mean temperature at −0.21 for the *young* chronology and 0.14 for the *old* chronology (Fig. 4). To avoid issues related to the segment length curse (Cook et al. 1995), the *old*, *young*, and instrumental data were detrended using a fixed 30-year spline for an additional comparison. This minimises potential low-frequency differences between the chronologies as positive trends are likely better preserved in the *old* chronology, while they are removed from the *young* chronology owing to the difference in segment length between the datasets. Similar to the analysis of the *young* and *old* ADS chronologies (Fig. 4), responses are contrary between the two sites for MND and comparable for MXD. Differences between the age classes are reduced for the 30spl TOR MXD but remain comparable for 30-year spline GAF MXD and MND to the ADS detrended chronologies (Fig. S6). The only difference is that for TOR MND, the *old* 30spl chronology correlates stronger than the *young* chronology compared to the ADS chronologies. From these comparisons, the influence of tree age on climate signal stability appears ambiguous at both sites and for both parameters, disallowing general statements concerning the inclusion of young samples. This finding is similar to the results of a network analysis in northern Fennoscandia, where more complex CSAEs were identified for *Pinus sylvestris* TRW than for MXD (Konter et al. 2016). Our results show that CSAEs not only differ between sites but can also vary between density parameters from one site, and more work is needed to untangle the significance of CSAEs, particularly with regard to improving climate reconstructions (Esper et al. 2008; Konter et al. 2016; Spelsberg et al. 2025).

### Declining temperature response

At both sites, the temperature correlations of the *old* chronologies over the past four decades (1981–2020) declined compared to the preceding four decades (1941–1980) although differences are only statistically significant for TOR MND and GAF MXD (Fig. 4). In the most recent period, the GAF *old* MXD chronology’s correlation with July and (JJA) mean temperature at 0.73 (0.79) surpasses those of the TOR *old* chronologies at 0.57 (0.77; Fig. 4). Overall, the differences between *old* and *young* MXD at either site are minor compared to the substantial variations of the TOR MND chronologies, where the signal deteriorates signifcantly between the two periods (from -0.69 (-0.60) to -0.14 (-0.29) for July (JJA)).

**Fig. 5:**
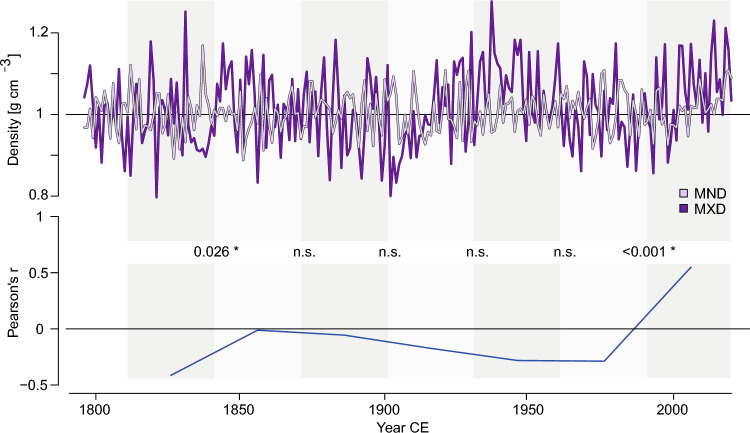
Comparison of detrended *full* TOR MND and MXD chronologies. Bottom panel shows the correlation calculated between successive 30-year intervals between the two chronologies. Numbers between panels indicate p-values for each period, n.s.; correlations not statistically significant at *p* < 0.05, *; correlations significant at *p* < 0.05

Nevertheless, the strong MND signal stands out for TOR, and correlations with MXD reveal two significant shifts over the last two centuries (also found between the *old* chronologies). Generally, the density parameters appear independent until a sudden change in the late twentieth century, after which they display a strong positive correlation (Fig. 5, for GAF see Fig. S7). Initially, the positive association between TOR MXD and MND contradicts their correlations with mean temperature, but suggests a negative relationship between the two. This recent shift indicates a profound change in the relationship between MND and MXD, coinciding with the diminishing correlation between MND and mean July temperature, without any indication of a change in the timing of the MND temperature response.

### Changes in seasonal response

**Fig. 6:**
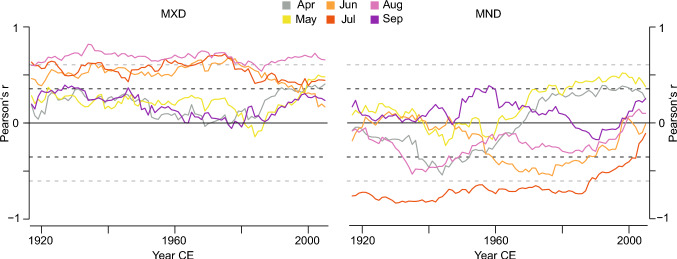
Temporal stability and seasonal fidelity of the TOR density climate signal. 31-year running correlations of MXD (left) and MND (right) against monthly warm season temperatures from 1902 to 2020. Dashed black lines indicate *p* < 0.05 significance and dashed gray lines after Bonferroni correction

The single-month correlations between the TOR MND and mean temperatures reveal no coherent pattern or temporal shift behind the weaker correlation in the last decades (Fig. 6). Beginning in the late 1970s, mean temperatures in April and May correlate positively for several decades before declining again towards the recent end of the chronology. June, August, and September display no significant influence or clear pattern. Only for July do the correlations remain stable from the beginning of the analyzed period (1902–2020) until a decline in the late 1980s, when the signal is completely lost (Fig. 6, for GAF see Fig. S8). Examining the seasonal fidelity of TOR MXD indicates an increasing importance of late spring and early summer temperatures (April, May) in a potential broadening of the growing season. Meanwhile, decreasing correlations with the mid-summer months June and July after 1980 could be an indication of the loss of temperature limitation under increasing temperatures. These weakening correlations of MXD with June and July contribute to the slight decline in JJA correlations. Shifting trends in the temperature data are eliminated as a cause for the deteriorating correlations, as the analysis of first-difference density and temperature data displays similar temporal patterns to those of the original *full* density chronologies and temperature data (Fig. S9). As there is no evidence to suggest that the influence of July on MND shifts to another month, it appears that MND is losing its connection with mean temperature (Fig. 6).

Historically, minimum (and earlywood) densities are not widely utilized in climate reconstructions, except for hydroclimatic relationships in arid sites (Camarero et al. 2017; Camarero and Hevia 2020) and, to a lesser extent, for temperature (Camarero et al. 2021). However, a possible biophysiological explanation could be that warm summers with adequate water supply, as observed at TOR, support rapid growth during relatively brief periods for cell maturation, resulting in less dense earlywood and correspondingly lower MND values (Björklund et al. 2020). There are several possible reasons for the changing response of MND to temperature. Changes in temperature and related climate variables could lead to an earlier onset of the growing season, potentially weakening the signal through an increase in the period and range of factors affecting wood formation. Other explanations previously proposed for the divergence between MXD and June to July temperatures, which are also applicable to MND, include reaching physiological threshold temperatures beyond which tree growth no longer responds to changes in temperature (D’Arrigo et al. 2004). This decline in the MND-temperature correlation also coincides with a shift in the relationship between MND and MXD in the 1980s. Evolving climate conditions may influence the changing association of MND and MXD, which could help identify past instances of similar climate change. An analysis of millennial-length MXD and corresponding MND records may uncover historical events where the correlation between MND and MXD changed, indicating comparable shifts in the climate sensitivity of tree growth, similar to those found in the TOR chronology.

## Conclusion

We analyzed two well-replicated chronologies of five density parameters to examine growth responses to climate variability and the influence of tree age. MXD serves as the strongest temperature proxy in both Scotland and Sweden. Including mixed sample ages does not affect these findings in any direction. There is a previously untapped potential in testing MND as a climate proxy, not only for precipitation but for temperature, accounting for over half the variance in mean July temperatures from 1901 to 1980. Despite this strong correlation, the relationship between MND and July temperatures weakens in more recent decades. Consequently, we recommend using already available density parameters to broaden our understanding of the impacts of climate change on tree growth and to help identify shifts in climate sensitivity, which might otherwise be overlooked if one focuses solely on the “best-performing” parameter. While such changes may vary by species and site, incorporating additional parameters requires minimal extra time and could provide valuable insights that have previously been ignored.

## Supplementary Information

Below is the link to the electronic supplementary material.

Supplementary file1 (DOCX 1962 kb)

## Data Availability

Data will be made available on request.
