# Supplementary material for: Beyond maximum density: multi-parameter insights into Scots pine climate sensitivity
Source: Trees (Berl West). 2025 Sep 22;39(5):101. doi: 10.1007/s00468-025-02681-3 (PMC12454479; doi:10.1007/s00468-025-02681-3)
Supplement: Supplementary file 1 — Supplementary file1 (DOCX 1962 kb) [file 468_2025_2681_MOESM1_ESM.docx]

Supplements

**Table S1**: Descriptive statistics for the ADS detrended chronologies by site and correlation of the density chronologies, asterisk indicating significance (*p* < 0.01).

|  | Parameter | EPS | Rbar | SD Rbar | Mean AR1 | SD AR1 | TRD | EWD | MND | LWD |
| --- | --- | --- | --- | --- | --- | --- | --- | --- | --- | --- |
| TOR | TRD | 0.97 | 0.56 | 0.19 | 0.26 | 0.19 |  |  |  |  |
|  | EWD | 0.96 | 0.48 | 0.23 | 0.37 | 0.22 | 0.74* |  |  |  |
|  | MND | 0.95 | 0.41 | 0.22 | 0.32 | 0.21 | 0.52* | 0.90* |  |  |
|  | LWD | 0.98 | 0.63 | 0.22 | 0.18 | 0.22 | 0.70* | 0.19* | -0.08 |  |
|  | MXD | 0.98 | 0.64 | 0.23 | 0.20 | 0.22 | 0.68* | 0.17* | -0.10 | 0.99* |
| GAF | TRD | 0.96 | 0.48 | 0.17 | 0.31 | 0.24 |  |  |  |  |
|  | EWD | 0.94 | 0.42 | 0.15 | 0.43 | 0.20 | 0.86* |  |  |  |
|  | MND | 0.92 | 0.34 | 0.14 | 0.39 | 0.22 | 0.74* | 0.94* |  |  |
|  | LWD | 0.96 | 0.45 | 0.19 | 0.34 | 0.17 | 0.64* | 0.36* | 0.24* |  |
|  | MXD | 0.96 | 0.46 | 0.20 | 0.34 | 0.17 | 0.60* | 0.31* | 0.17* | 0.99* |


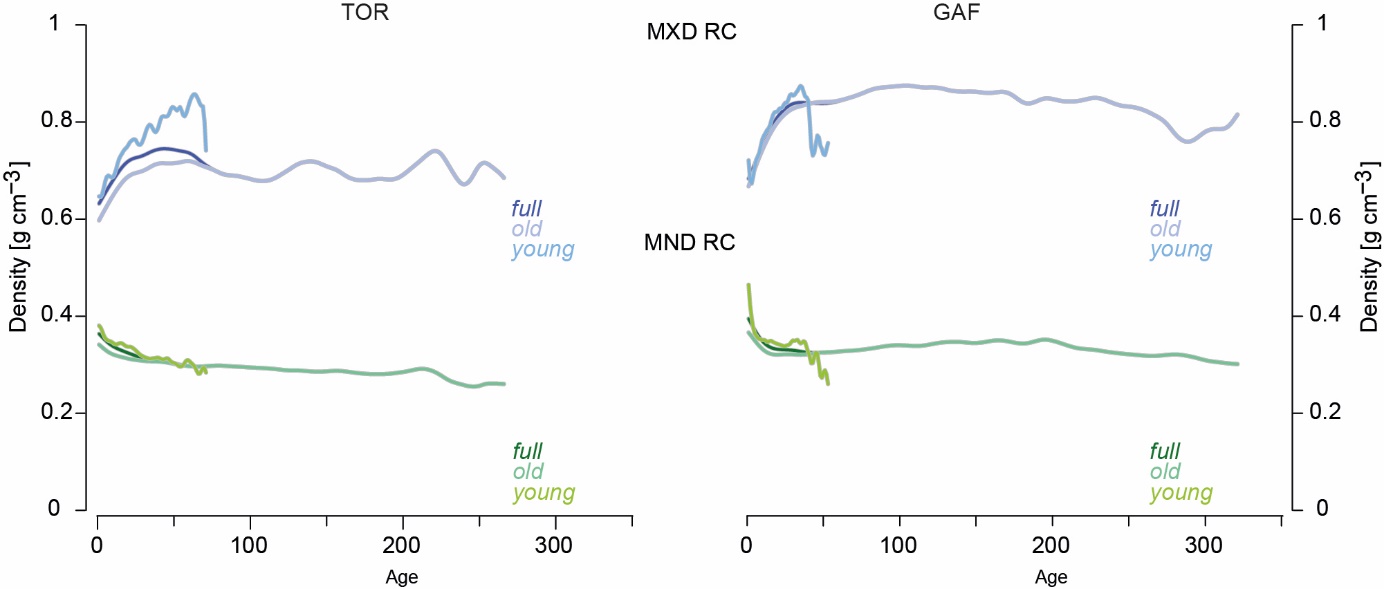


**Fig. S1**: Regional curves for TOR on the left and GAF on the right with MND (green hues) and MXD (blue hues) for the *full*, *old,* and *young* chronologies.


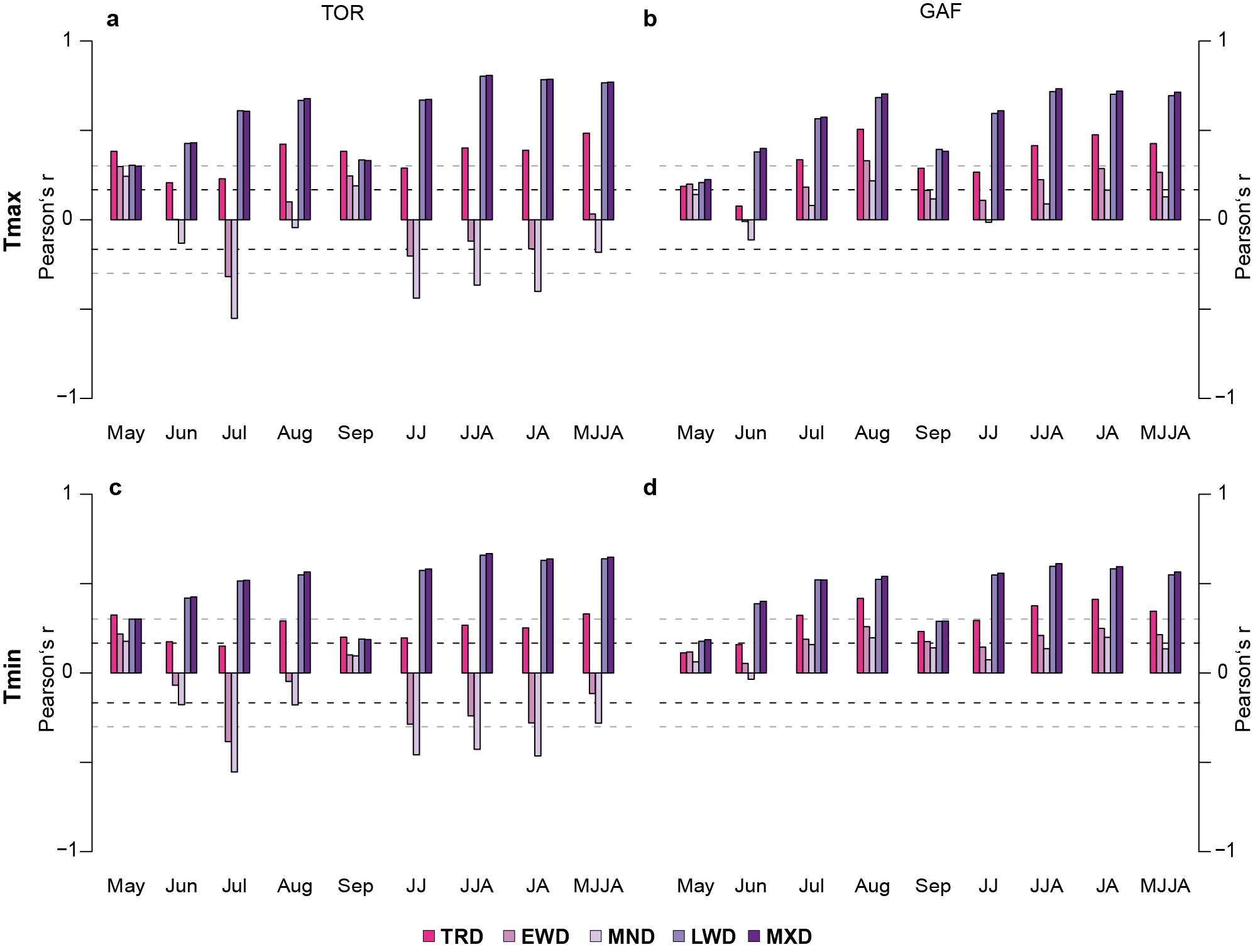


**Fig. S2** Temperature sensitivity of tree-ring density parameters. **a-b**, Correlations of the *full* TOR and GAF chronologies with maximum monthly and seasonal temperatures from 1902-2020. Dashed black lines indicate *p* < 0.05 significance, and dashed grey lines indicate significance adjusted using Bonferroni correction. **c-d**, Correlations of the *full* TOR and GAF chronologies with minimum monthly and seasonal temperatures from 1902-2020. Dashed black lines indicate *p* < 0.05 significance, and dashed grey lines indicate significance adjusted using Bonferroni correction


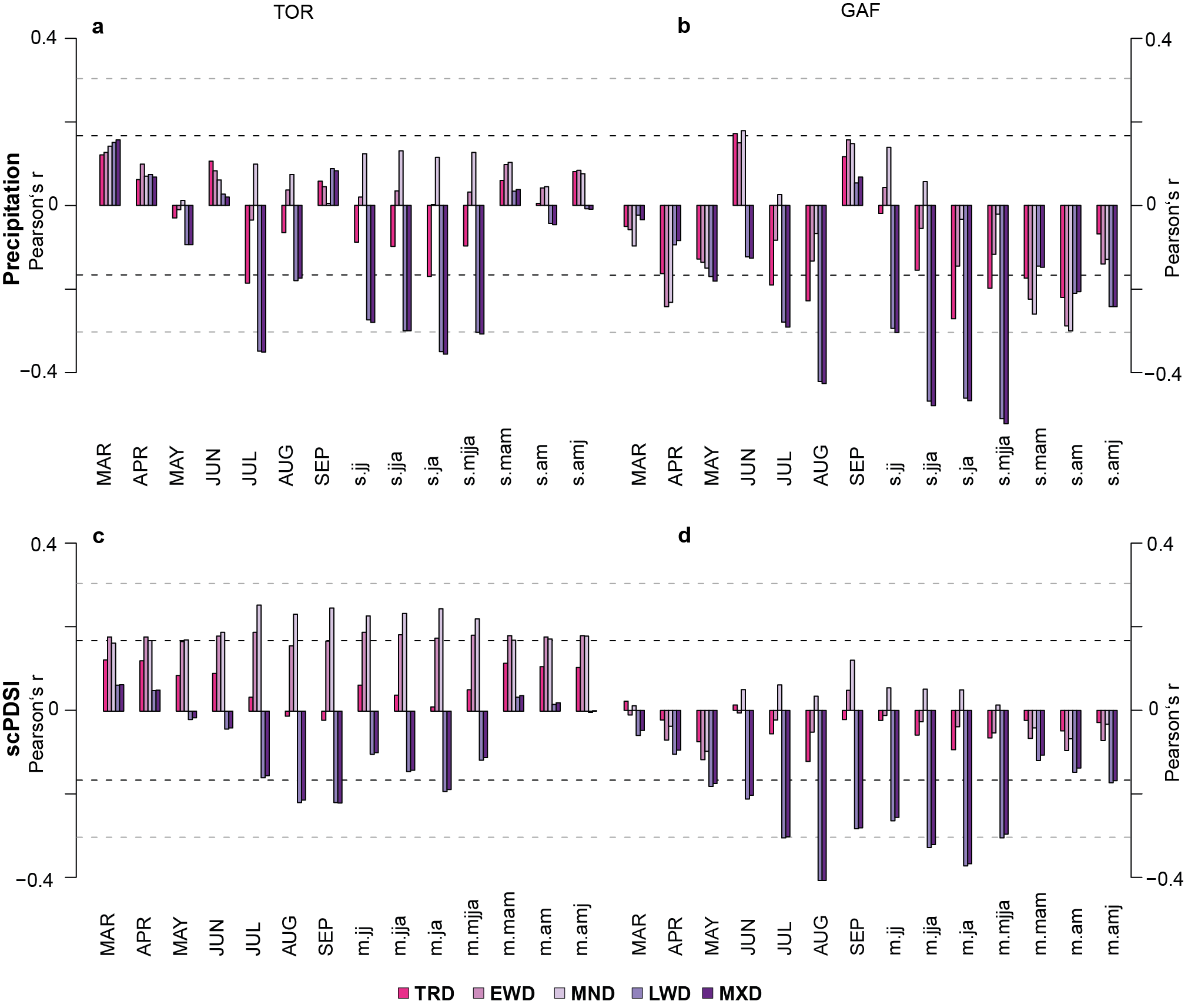


**Fig. S3** Climate response of density parameters. **a-b**, Correlations of the *full* TOR and GAF chronologies with monthly and seasonal (s = sum) precipitation from 1902-2020. Dashed black lines indicate *p* < 0.05 significance, and dashed grey lines indicate significance adjusted using Bonferroni correction. **c-d**, Correlations of the *full* TOR and GAF chronologies with monthly and seasonal (m = mean) scPDSI from 1902-2020. Dashed black lines indicate *p* < 0.05 significance, and dashed grey lines indicate significance adjusted using Bonferroni correction


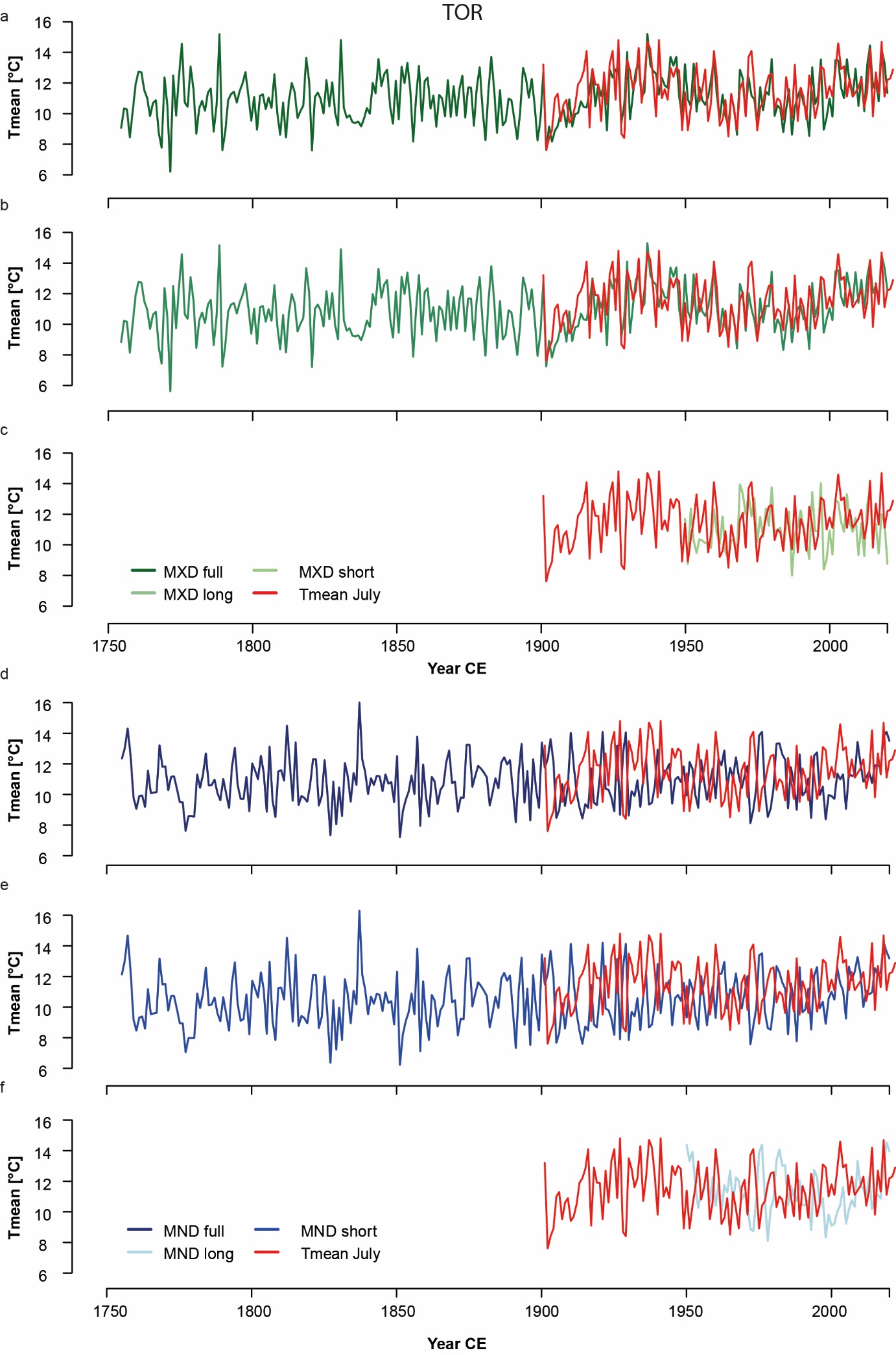


**Fig S4**: July mean temperature and TOR MXD ADS full (a), long (c) and short (d) and MND ADS full (d), long (e) and short (f) chronologies, all scaled to 1981-2020.


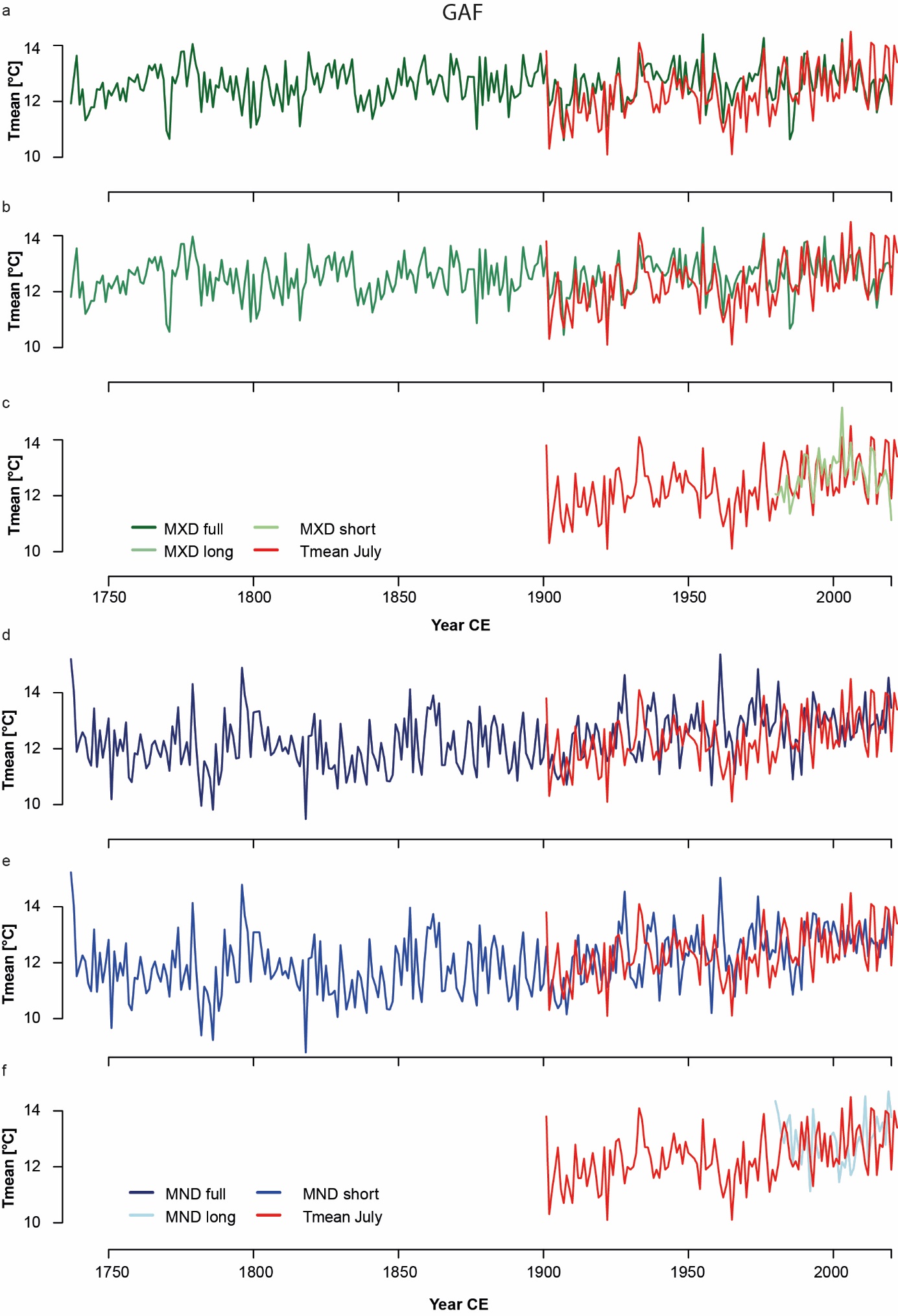


**Fig S5**: July mean temperature and GAF MXD ADS full (a), long (c) and short (d) and MND ADS full (d), long (e) and short (f) chronologies, all scaled to 1981-2020.


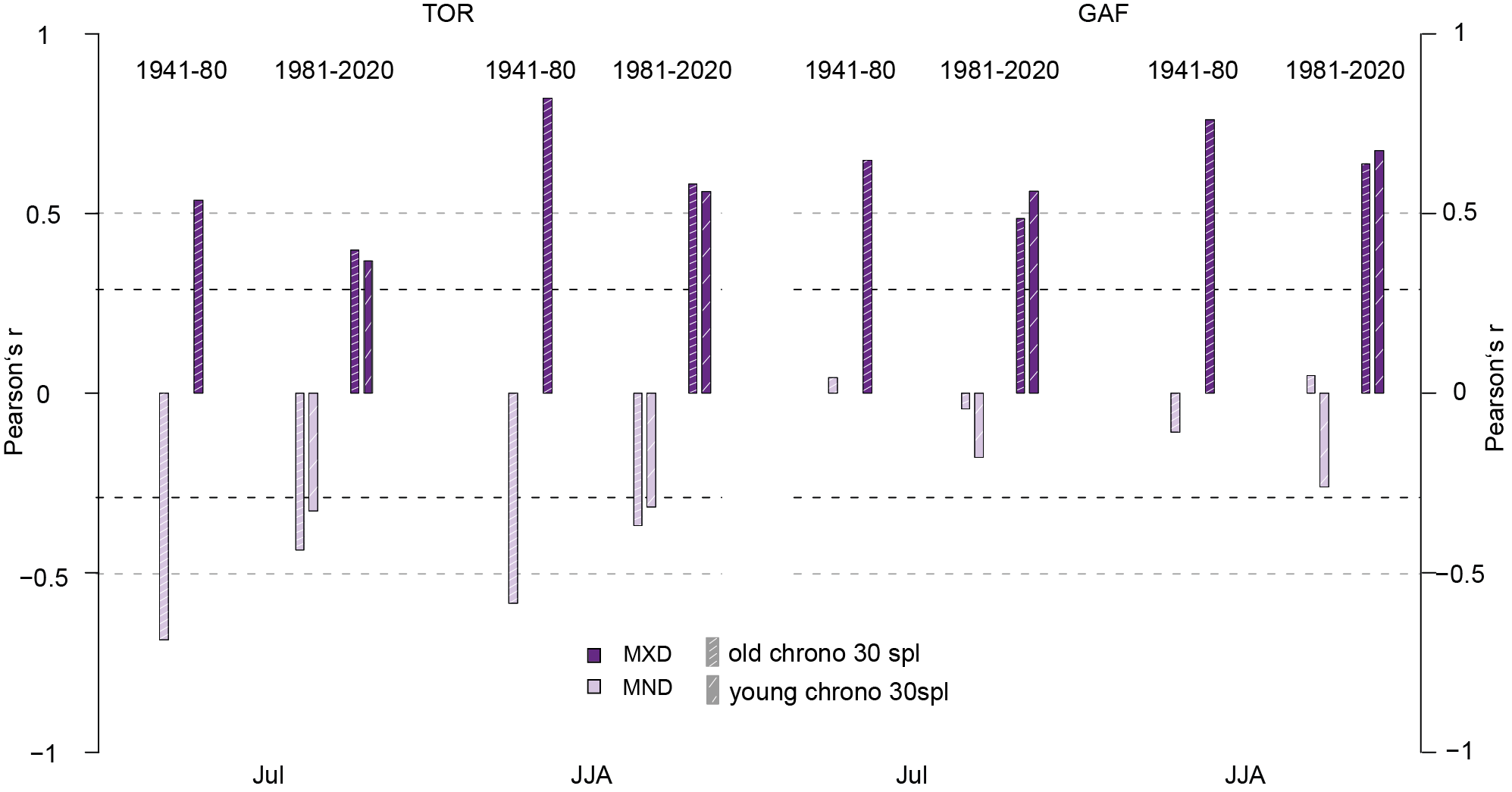


**Fig. S6** Influence of tree age on climate signals after 30-year spline detrending of chronologies and instrumental data. Correlation of MND (light pink) and MXD (purple) *full* (blank), *old* (tight hatch) and *young* (loose hatch) chronologies with July and June-July-August (JJA) temperatures. Dashed black lines indicate *p* < 0.05 significance and dashed grey lines after adjusted using the Bonferroni correction


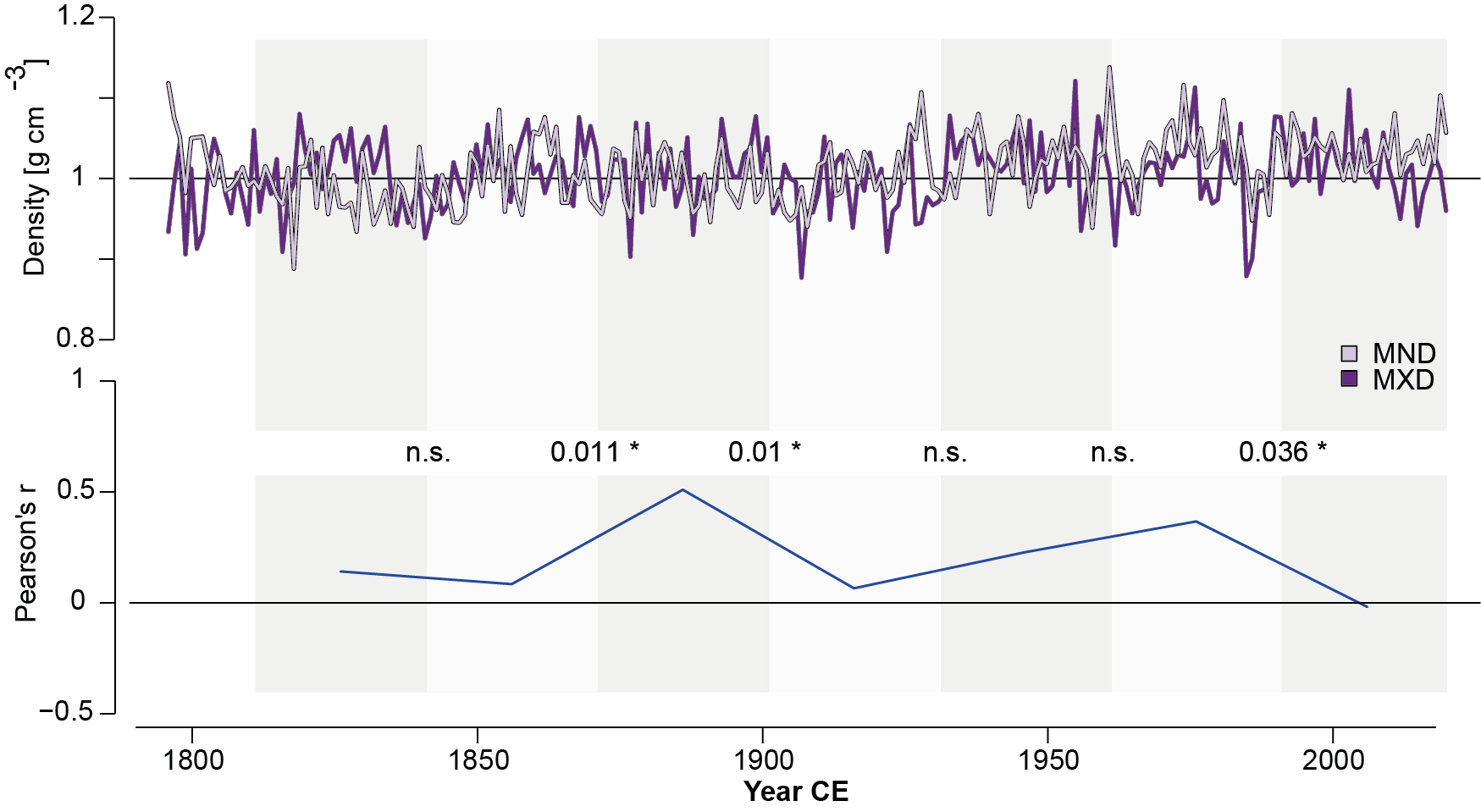


**Fig. S7** Comparison of *full* GAF MND and MXD detrended chronologies (top panel). Correlation of 30-year intervals of the *full* GAF MND and MXD chronologies (in blue) and p-values (black numbers) assessing the significance of changes between successive 30-year intervals, asterisk indicating significant (*p* < 0.05) change between two periods (bottom panel)


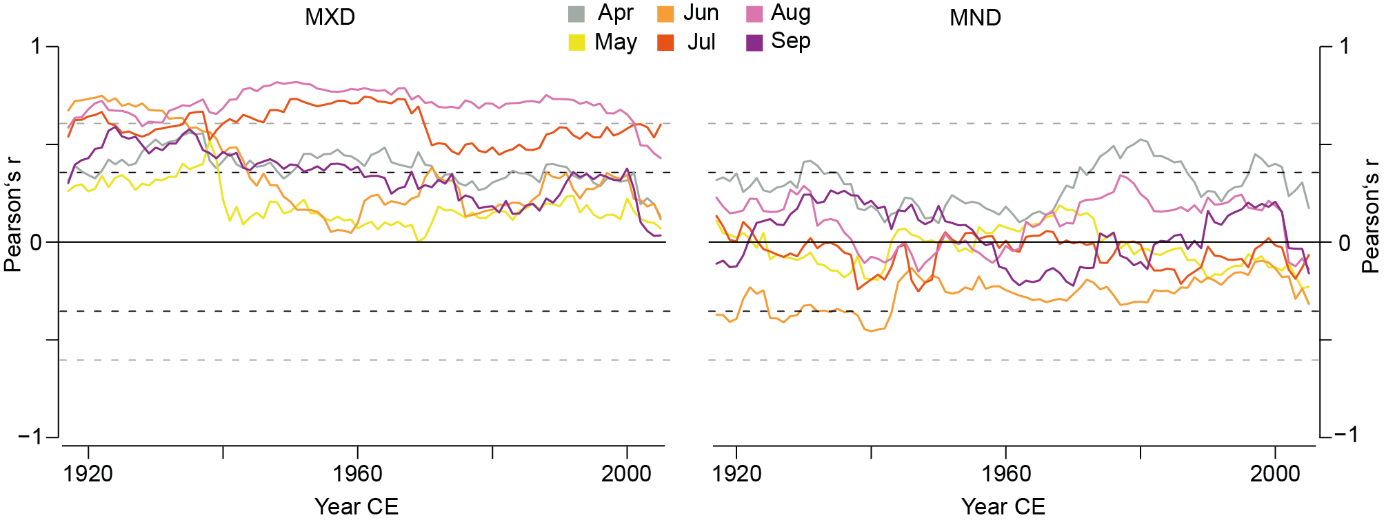


**Fig. S8** Seasonal fidelity of *full* GAF MXD and MND temperature correlation. Moving correlation (31-year window moved by 1 year) of MXD (top) and MND (bottom) with a mean temperature of single months from 1902–2020. Dashed black lines indicate *p* < 0.05 significance, and dashed grey lines indicate significance adjusted using Bonferroni correction


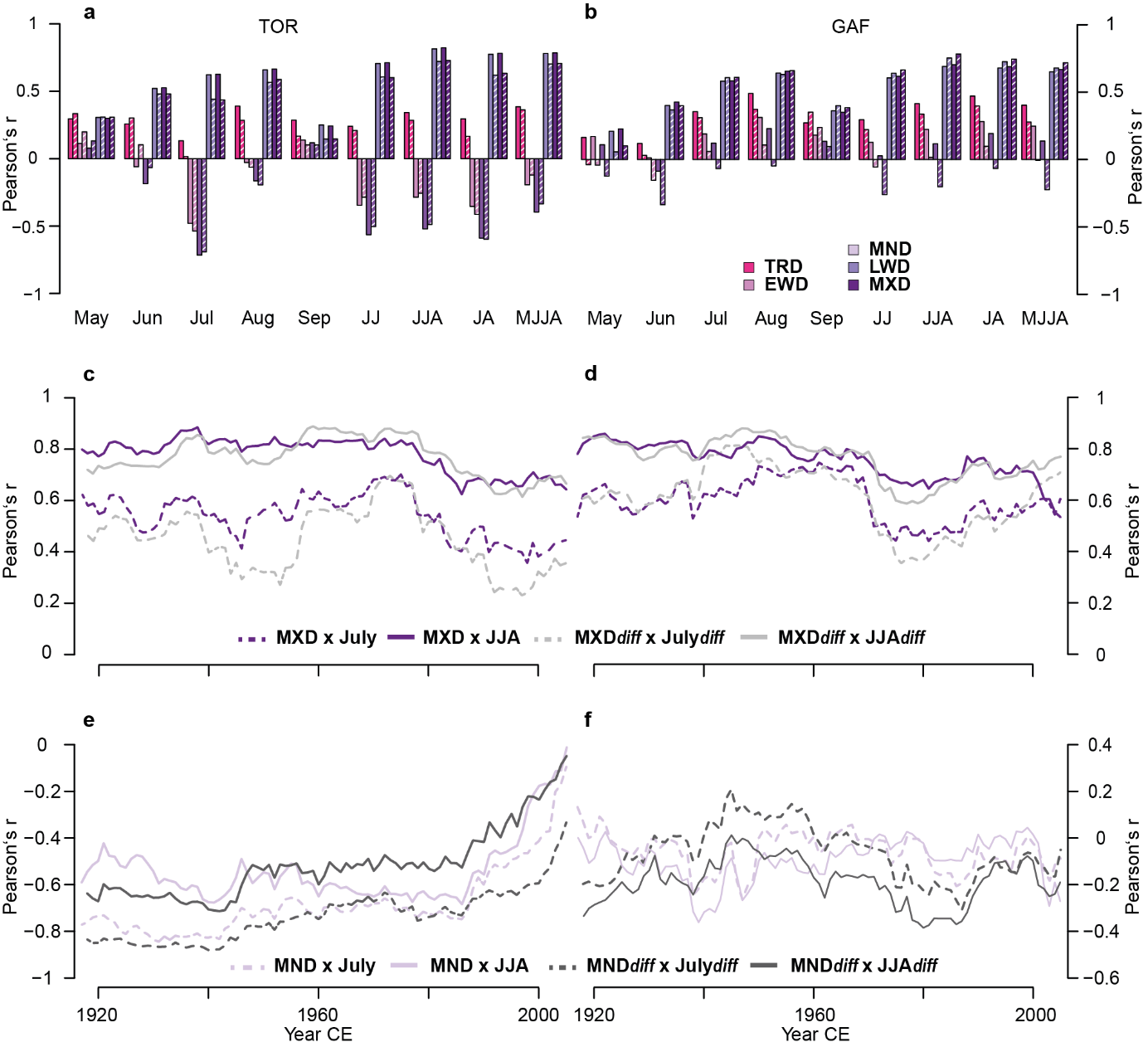


**Fig. S9** Temperature sensitivity of density parameters. **a**. Monthly and seasonal correlations of the *full* TOR original (first difference (hatched)) density chronologies and mean temperature from 1902 to 2020 (1903 to 2020). **b**. Monthly and seasonal correlations of the GAF original (first difference (hatched)) density chronologies and mean temperature from 1902 to 2020 (1903 to 2020). **c**. Moving correlation (31-year windows shifted by 1 year) of the TOR MXD original (purple) and MXD first difference (grey) with mean July temperature (dashed line), and mean June-July-August temperature (solid line) from 1902 (1903) to 2020. **d**. Moving correlation (31-year windows shifted by 1 year) of the *full* GAF MXD original (purple) and MXD first difference (grey) with mean July temperature (dashed line), and mean June-July-August temperature (solid line) from 1902 (1903) to 2020. **e**. Moving correlation (31-year windows shifted by 1 year) of the TOR MND original (light pink) and MXD first difference (grey) with mean July temperature (dashed line), and mean June-July-August temperature (solid line) from 1902 (1903) to 2020. **f**. Moving correlation (31-year windows shifted by 1 year) of the GAF MND original (light pink) and MXD first difference (grey) with mean July temperature (dashed line), and mean June-July-August temperature (solid line) from 1902 (1903) to 2020

**Table S2**: Descriptive statistics for the ADS detrended *young* chronologies by site.

|  | Parameter | EPS | Rbar | SD Rbar | Mean AR1 | SD AR1 |
| --- | --- | --- | --- | --- | --- | --- |
| TOR | TRD | 0.931 | 0.475 | 0.198 | 0.228 | 0.222 |
|  | EWD | 0.929 | 0.416 | 0.209 | 0.294 | 0.227 |
|  | MND | 0.943 | 0.548 | 0.239 | 0.062 | 0.194 |
|  | LWD | 0.922 | 0.374 | 0.219 | 0.297 | 0.208 |
|  | MXD | 0.945 | 0.532 | 0.264 | 0.099 | 0.200 |
| GAF | TRD | 0.848 | 0.343 | 0.182 | 0.147 | 0.234 |
|  | EWD | 0.822 | 0.326 | 0.163 | 0.299 | 0.201 |
|  | MND | 0.817 | 0.301 | 0.196 | 0.259 | 0.200 |
|  | LWD | 0.846 | 0.267 | 0.144 | 0.255 | 0.226 |
|  | MXD | 0.806 | 0.302 | 0.199 | 0.271 | 0.208 |
